# RNA-Sequencing-Based Transcriptomic Score with Prognostic and Theranostic Values in Multiple Myeloma

**DOI:** 10.3390/jpm11100988

**Published:** 2021-09-30

**Authors:** Elina Alaterre, Veronika Vikova, Alboukadel Kassambara, Angélique Bruyer, Nicolas Robert, Guilhem Requirand, Caroline Bret, Charles Herbaux, Laure Vincent, Guillaume Cartron, Olivier Elemento, Jérôme Moreaux

**Affiliations:** 1 Institute of Human Genetics, UMR 9002 CNRS-UM, 34395 Montpellier, France; elina.alaterre@igh.cnrs.fr (E.A.); vvikova78@gmail.com (V.V.); alboukadel.kassambara@gmail.com (A.K.); a.bruyer@diag2tec.com (A.B.); c-bret@chu-montpellier.fr (C.B.); c-herbaux@chu-montpellier.fr (C.H.); 2 Diag2Tec, 34395 Montpellier, France; 3 Department of Biological Hematology, CHU Montpellier, 34395 Montpellier, France; nicolas.robert@igh.cnrs.fr (N.R.); guilhem.requirand@igh.cnrs.fr (G.R.); 4 UFR de Médecine, University of Montpellier, 34003 Montpellier, France; g-cartron@chu-montpellier.fr; 5 Department of Clinical Hematology, CHU Montpellier, 34395 Montpellier, France; l-vincent@chu-montpellier.fr; 6 IGMM, UMR CNRS-UM 5535, 34090 Montpellier, France; 7 Caryl and Israel Englander Institute for Precision Medicine, Weill Cornell Medicine, New York, NY 10021, USA; ole2001@med.cornell.edu; 8 IUF, Institut Universitaire de France, 75005 Paris, France

**Keywords:** multiple myeloma, risk stratification, gene expression profiling, precision medicine

## Abstract

Multiple myeloma (MM) is the second most frequent hematological cancer and is characterized by the clonal proliferation of malignant plasma cells. Genome-wide expression profiling (GEP) analysis with DNA microarrays has emerged as a powerful tool for biomedical research, generating a huge amount of data. Microarray analyses have improved our understanding of MM disease and have led to important clinical applications. In MM, GEP has been used to stratify patients, define risk, identify therapeutic targets, predict treatment response, and understand drug resistance. In this study, we built a gene risk score for 267 genes using RNA-seq data that demonstrated a prognostic value in two independent cohorts (n = 674 and n = 76) of newly diagnosed MM patients treated with high-dose Melphalan and autologous stem cell transplantation. High-risk patients were associated with the expression of genes involved in several major pathways implicated in MM pathophysiology, including interferon response, cell proliferation, hypoxia, IL-6 signaling pathway, stem cell genes, MYC, and epigenetic deregulation. The RNA-seq-based risk score was correlated with specific MM somatic mutation profiles and responses to targeted treatment including EZH2, MELK, TOPK/PBK, and Aurora kinase inhibitors, outlining potential utility for precision medicine strategies in MM.

## 1. Introduction

Multiple myeloma (MM) is an incurable malignant plasma cell disorder characterized by strong genetic heterogeneity impacting treatment response and clinical outcomes [1,2,3]. Pre-clinical studies have identified a compendium of mechanisms associated with MM cell treatment resistance. Given this heterogeneity, one of the current challenges is to precisely predict survival and treatment response according to patient molecular characteristics in order to develop personalized medicine. Gene expression profiling (GEP) has created major insights in both the understanding and clinical management of this disease. Indeed, analysis of patient transcriptomic data at diagnosis has highlighted substantial molecular heterogeneity between patients that is characterized by distinct gene signatures and that is associated with clinical outcomes [4]. Several GEP-based signatures predicting prognosis have been reported, including UAMS (70 genes) [5], HOVON-65/GMMG-HD4 (92 genes) [6], and IFM (15 genes) [7]. These signatures identify newly diagnosed MM patients characterized by a poor outcome after treatment with high-dose melphalan and autologous stem cell transplantation. We previously built a three-group risk prediction model for overall survival (OS), which was called RS to represent the risk score [8]. Other signatures with prognostic value have been connected to the biological mechanisms involved in MM progression [9,10,11,12]. GEP-derived signatures that predict response or resistance to proteasome inhibitors (PIs) [13,14,15,16], melphalan [17], IMiDs [18], HDACi [19], DNMTi [20], EZH2 inhibitors [21], or kinase inhibitors [22] have also been reported. Thus, in MM, GEP is useful for predicting prognosis and screening for drug resistance biomarkers with potential benefits for clinical management. However, most previous studies have been conducted using microarray technology, which entails the analysis of a limited number of genes and potential artefacts due to incorrect probe design, genetic variation, and inefficient hybridization. Microarrays are being phased out and replaced by RNA sequencing (RNAseq), which overcomes many of the limitations associated with microarrays. It is expected that RNAseq will enable the discovery of new prognostic markers, associations with drug response, and the mechanisms underlying MM pathophysiology. To that aim, we used RNAseq and clinical data for 674 newly diagnosed patients treated with standard therapy from the Multiple Myeloma Research Foundation CoMMpass study to build a 267-gene risk score. Validation was performed using a cohort of 76 newly diagnosed MM patients from our center. The RNA-seq-based risk score demonstrated prognostic value in the two independent cohorts of newly diagnosed MM patients. High-risk patients were characterized by the expression of genes involved in several major pathways implicated in MM pathophysiology, including cell proliferation, MYC pathways, and epigenetic regulation. Additionally, our RNAseq-based risk score was associated with specific MM mutational profiles and responses to targeted treatment, underlining the potential utility of precision medicine strategies in MM.

## 2. Materials and Methods

### 2.1. Gene Expression Profiling

We used the publicly available gene expression profiling RNA-seq data of newly diagnosed MM patients from the Multiple Myeloma Research Foundation’s (MMRF) CoMMpass study (https://research.themmrf.org/ (28 September 2021), release IA12). Concerning the validation cohort, bone marrow samples were collected after obtaining written informed consent from the patients in accordance with the Declaration of Helsinki and after receiving institutional research board approval from Montpellier University Hospital. Bone marrow samples were collected from 76 newly diagnosed patients treated with high-dose melphalan (HDM) and autologous stem cell transplantation (ASCT), and this cohort was termed the Montpellier cohort. The bone marrow of patients presenting with previously untreated MM at the University Hospital of Montpellier was obtained after obtaining written informed consent from the patients in accordance with the Declaration of Helsinki and according to the IRB agreement and based on the approval of the Montpellier University Hospital Centre for Biological Resources (DC-2008-417). Patient MM cells (MMCs) were purified using anti-CD138 MACS microbeads (Miltenyi Biotec, Bergisch Gladbach, Germany), and their gene expression profiles (GEP) were obtained using RNA-sequencing. The RNA sequencing (RNA-seq) library preparation was completed with 150 ng of input RNA using the Illumina TrueSeq Stranded mRNA Library Prep Kit. Paired-end RNA-seq was performed with an Illumina NextSeq sequencing instrument (Helixio, Clermont-Ferrand, France). RNA-seq read pairs were mapped to the reference human GRCh37 genome using the STAR aligner [23].

### 2.2. Statistical Analyses

All of the statistical analyses were performed with the R statistics software (version 3.2.3; available from https://www.r-project.org) (28 September 2021) and with R packages developed by the BioConductor project (available from https://www.bioconductor.org/) (28 September 2021) [24]. The expression level of each gene was summarized and normalized using the DESeq2 R/Bioconductor package [25]. Differential expression analysis was performed using the DESeq2 pipeline [25]. *p*-values were adjusted to control the global FDR across all comparisons with the default option of the DESeq2 package. Genes were considered differentially expressed with an adjusted *p*-value < 0.05 and a fold change > 1.5. For the Montpellier cohort, Affymetrix U133P chips were also used, as previously described [22,26], to analyze GEP and to calculate previously published risk scores, including the RS score [8], UAMS HRS score [5], and IFM score [7].

### 2.3. Multiple Myeloma Cell Lines

XGs human myeloma cell lines (HMCLs) were obtained as previously described [27,28]. AMO-1, LP1, L363, OPM2, MOLP2, MOLP8, Lopra, and SKMM2 were purchased from DSMZ (Braunsweig, Germany), and RPMI8226 was purchased from ATCC (American Tissue Culture Collection, Rockville, MD, USA). JJN3 was kindly provided by Dr. Van Riet (Bruxelles, Belgium), and was provided MM1S by Dr. S. Rosen (Chicago, USA). HMCLs were authenticated according to their short tandem repeat profiling and their gene expression profiling using Affymetrix U133 plus 2.0 microarrays deposited in the ArrayExpress public database under accession numbers E-TABM-937 and E-TABM-1088.

### 2.4. Drug Response Analyses

HMCLs were cultured in RPMI-1640 medium (Gibco, Thermo Fisher Scientific, Waltham, MA, USA) supplemented with fetal bovine serum (FBS, Eurobio, Les Ulis, France) (10%) and interleukin 6 (IL6, Peprotech, Rocky Hill, New Jersey, USA) for XG cell lines. We evaluated the sensitivity of the cell lines to twenty-two drugs, including EZH2 inhibitor (EPZ-6438), Aurora kinase inhibitor (MLN8237), MELK inhibitor (OTSSP167), and PBK inhibitor (Hi-TOPK-032). For a given drug, the HMCLs were treated with different concentrations. The IC_50_ was determined at day 4 using the CellTiter-Glo assay (Promega, Madison, Wisconsin, USA), as previously described [28], with the exception of EPZ-6438. HMCLs were cultured for 8 days with (treatment) and without (control) EPZ-6438. Cell concentration and viability were assessed using the trypan blue dye exclusion test.

### 2.5. EZH2 Inhibition in Primary MM Cells from Patients

Mononuclear cells from tumor samples of seven patients with previously untreated MM from the Montpellier cohort were cultured for 8 days in the presence of IL-6 (2ng/mL) with and without 1µM EPZ-6438. At day 8 of the culture period, the count of viable MMC was determined using CD138 staining by flow cytometry.

## 3. Results

### 3.1. RNA-Seq-Based Gene Risk Score in Multiple Myeloma

**Figure 1:**
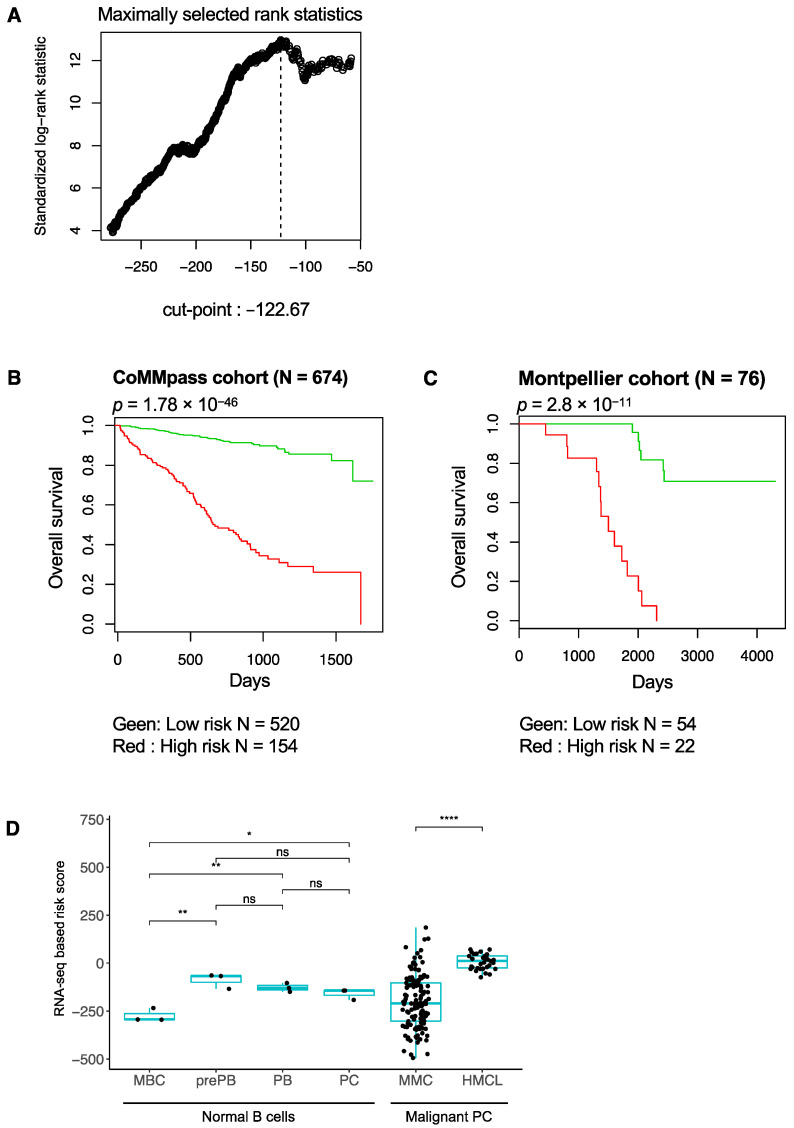
Prognostic value of the RNA-seq-based risk score. (**A**) Maxstat defined cut-point and prognostic value of RNA-seq-based risk score in CoMMpass cohort (n = 674 MM patients). The RNA-seq risk score split MM patients into low- and high-risk groups in two independent cohorts: (**B**) CoMMpass and (**C**) Montpellier (n = 76 MM patients) (OS, Kaplan–Meier curves). (**D**) RNA-seq-based risk score value calculated in normal human B to plasma cell differentiation and in malignant plasma cells from MM patients (n = 129) and HMCLs (n = 33). Wilcoxon test. NS: nonsignificant, * *p*-value < 0.05, ** *p*-value < 0.01, **** *p*-value < 0.0001.

We used the gene expression profiling (GEP) data of 674 newly diagnosed MM patients from the Multiple Myeloma Research Foundation’s (MMRF) CoMMpass study. We also performed the RNA sequencing of purified MMC from 76 newly diagnosed MM patients treated by high dose therapy (HDT) and autologous stem cell transplantation. Using the Maxstat R function and the Benjamini–Hochberg multiple testing correction, 267 genes were found to have a prognostic value for overall survival (OS) (adjusted *p* value < 0.05) in the CoMMpass cohort of patients with previously untreated MM. These 267 prognostic genes comprised 142 genes associated with a poor outcome in MM and 97 genes associated with high expression related to a good prognostic value. The 267 genes were used to build an RNA-seq-based risk score. The RNA-seq-based risk score is defined by the sum of the beta coefficient derived from the Cox model for each prognostic gene weighted by -1 or +1 according to the MMC gene expression above or below the Maxstat defined cutpoint [29]. Patients from the CoMMpass cohort were ranked according to increased RNA-seq-based risk scores, and the Maxstat algorithm was used to find the cutoff associated with the maximum difference in the OS (Figure 1A). The RNA-seq-based risk score split patients into a high-risk group (22.8%) and a low-risk group (77.2%) in the CoMMpass cohort (*p*-value = 1.7 × 10^−4^^6^) (Figure 1B). The prognostic value of the RNA-seq-based risk score was validated in the Montpellier cohort (*p*-value = 2.8 × 10^−11^) (Figure 1C). When applied to a non-disease dataset, i.e., the GEP profiles from a normal B to plasma cell differentiation model, significantly higher score values were identified in pre-plasmablast, plasmablast, and plasma cell stages compared to in the memory B cell stage (Figure 1D). These data demonstrate that the RNA-seq-based risk score is not a hallmark of proliferation since no significant difference was found between the proliferating pre-plasmablasts and the non-proliferating plasma cells (Figure 1D). Furthermore, the RNA-seq-based risk score was not correlated with plasma cell labeling index (PCLI—percentage of MM cells in S phase [30]) at diagnosis in the Montpellier cohort (Supplementary Figure S1). Significantly higher score values were also observed in the HMCLs compared to in the MM cells of patients, indicating that a high score is associated with MM progression (Figure 1D). Altogether, these data highlight that the RNA-seq-based risk score can identify newly diagnosed high-risk MM patients in independent cohorts.

### 3.2. High-Risk MM Patients Identified with the RNA-Seq-Based Risk Score Are Characterized by Enrichment of Genes Related to Cell Proliferation, Growth Factor Signaling, MYC Pathway and Epigenetic Deregulation

**Figure 2:**
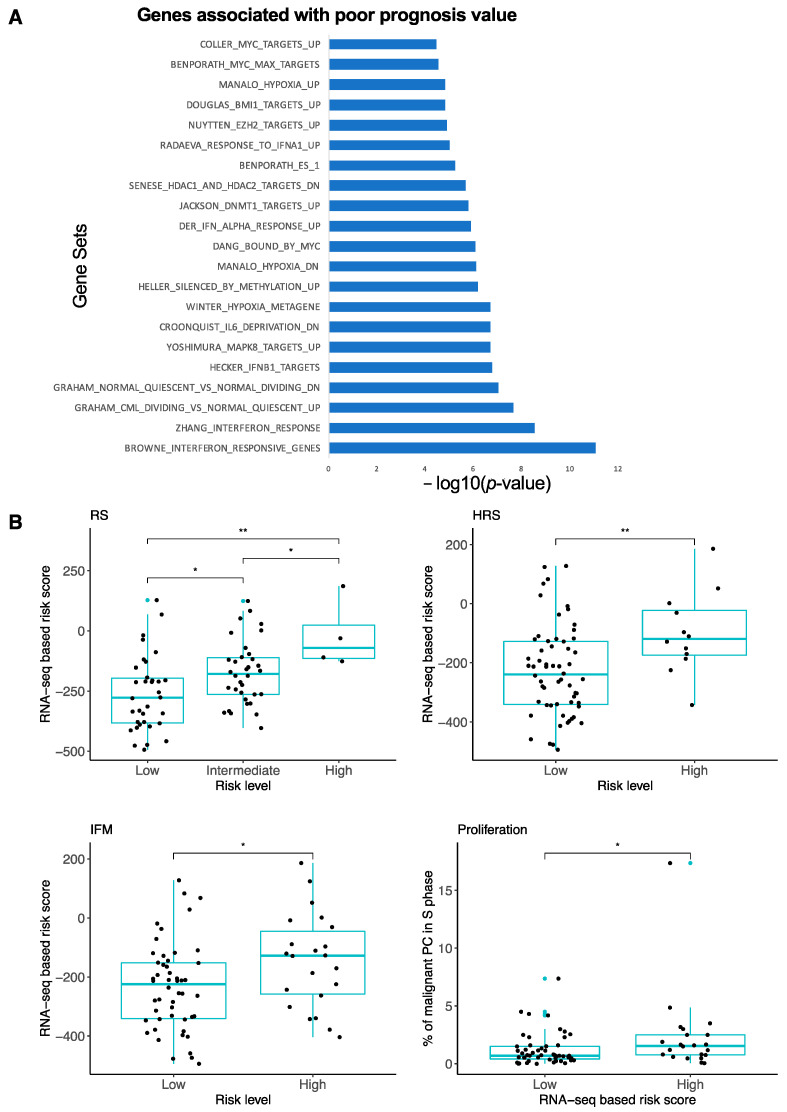
Characterization of RNA-seq-based risk score. (**A**) Gene set enrichment analysis of RNA-seq-based risk score genes associated with poor prognosis. DN: downregulated; UP: upregulated. A complete description of all of the molecular signatures presented is available in the GSEA molecular signatures database (https://www.gsea-msigdb.org/gsea/msigdb/index.jsp) (28 September 2021). (**B**) Comparison of RNA-seq-based risk score with Affymetrix GEP-based risk scores (RS, HRS, IFM) and proliferation. Wilcoxon test. ns: nonsignificant, * *p*-value < 0.05, ** *p*-value < 0.01.

GSEA [31,32] analyses revealed that the genes associated with a poor prognostic value were significantly enriched in the genes related to interferon response, cell proliferation, hypoxia, IL-6 signaling pathway, interferon (IFN) response, stem cell genes, MYC, and epigenetic deregulation (Figure 2A). Among the epigenetics-related genes enriched in high-risk MM patients, EZH2 targets, HDAC targets, and DNA methylation target genes were identified. We next investigated the RNA-seq-based risk score value distribution according to the Affymetrix GEP-based risk scores described in MM. The RNA-seq-based risk score values were significantly higher in patients who were defined as high-risk based on their RS score [8], UAMS HRS score [5], and IFM score [7]. Furthermore, high-risk RNA-seq-based score patients demonstrated a significant increase in the percentage of proliferating MM cells (median = 0.7%; range: 0–7.3%) compared to the low-risk group (median = 1.55%, range: 0–17.3%) (Figure 2B).

### 3.3. Association between RNA-Seq-Based Risk Score and Mutations in MM

**Figure 3:**
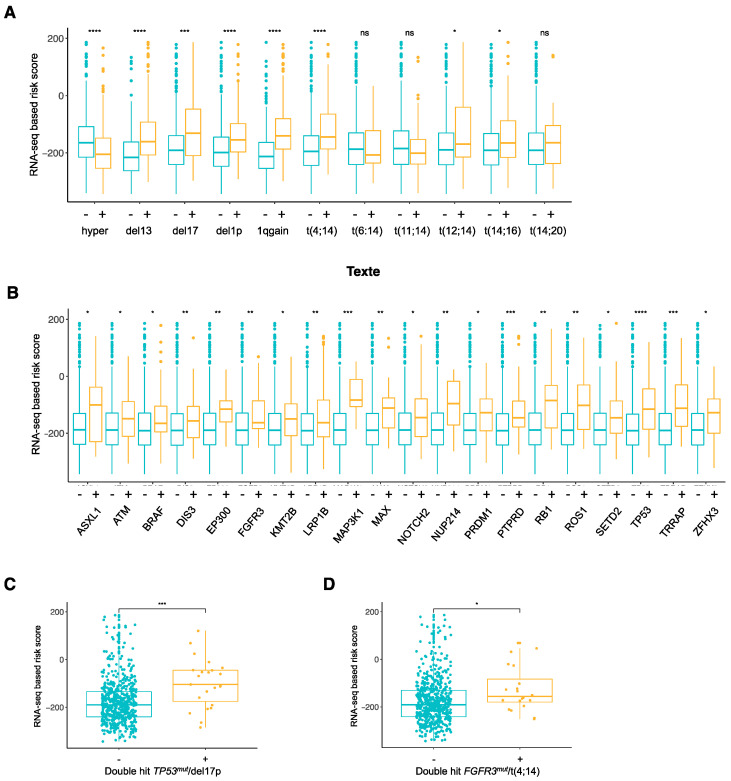
Association between RNA-seq-based risk scores and cytogenetic abnormalitie subgroups and mutations in MM patients from the CoMMpass cohort. (**A**) RNA-seq-based risk score in cytogenetic abnormality subgroups. Cytogenetic abnormality subgroups were composed of hyperdyploid (without: n = 240, with: n = 331), del(13p) (without: n = 307, with: n = 266), del(17p) (without: n = 523, with: n = 50), del(1p) (without: n = 455, with: n = 118), 1q gain (without: n = 378, with: n = 195), groups of patients and patients harboring t(4;14) (without: n = 515, with: n = 73), t(6;14) (without: n = 572, with: n = 16), t(11;14) (without: n = 461, with: n = 127), t(12;14) (without: n = 564, with: n = 24), t(14;16) (without: n = 532, with: n = 56), and t(14;20) (without: n = 543, with: n = 45) translocations. (**B**) Comparison of RNA-seq-based risk scores according to the status of recurrent mutated genes in MM: ASXL1 (−: n = 616, +: n = 15), ATM (−: n = 586, +: n = 45), BRAF (−: n = 576, +: n = 55), DIS3 (−: n = 554, +: n = 77), EP300 (−: n = 614, +: n = 17), FGFR3 (−: n = 592, +: n = 39), KMT2B (−: n = 555, +: n = 76), LRP1B (−: n = 532, +: n = 99), MAP3K1 (−: n = 620, +: n = 11), MAX (−: n = 599, +: n = 32), NOTCH2 (−: n = 596, +: n = 35), NUP214 (−: n = 617, +: n = 14), PRDM1 (−: n = 604, +: n = 27), PTPRD (−: n = 572, +: n = 59), RB1 (−: n = 613, +: n = 18), ROS1 (−: n = 605, +: n = 26), SETD2 (−: n = 597, +: n = 34), TP53 (−: n = 579, +: n = 52), TRRAP (−: n = 605, +: n = 26), and ZFHX3 (−: n = 592, +: n = 39). (**C**) Comparison of the RNA-seq-based risk score values in MM patients with (n = 23) and without (n = 546) double-hit TP53^mut^/del(17p). (**D**) Comparison of the RNA-seq-based risk score values in MM patients with (n = 20) and without (n = 562) double-hit FGFR3^mut^/t(4;14). Wilcoxon test. ns: nonsignificant, * *p*-value < 0.05, ** *p*-value < 0.01, *** *p*-value < 0.001, **** *p*-value < 0.0001.

We then analyzed the relationship between the RNA-seq-based risk score distribution, cytogenetic abnormalities, and mutational status in the CoMMpass cohort. The RNA-seq-based risk score values were significantly higher in MM patients with del(13q), del(17p), del(1p), 1qgain, t(4;14), t(12;14), and t(14,16) groups (Figure 3A). The RNA-seq-based risk score values of the MM patients were also evaluated according to the status of the most frequent mutations identified in HMCLs [28]. The RNA-seq-based risk scores were significantly higher in the MM patients and were characterized by more ASXL1, ATM, BRAF, DIS3, EP300, FGFR3, KMT2B, LRP1B, MAP3K1, MAX, NOTCH2, NUP214, PRDM1, PTPRD, RB1, ROS1, SETD2, TP53, TRRAP, and ZFHX3 mutations compared to the unmutated MM patients (Figure 3B). Interestingly, the RNA-seq-based risk scores were significantly higher in MM patients harboring double-hit TP53^mut^/del(17p) or FGFR3^mut^/t(4;14) compared to MM patients without double-hit (Figure 3C–D). The prognostic value of the RNA-seq-based risk score was then compared with the molecular subgroups and mutations mentioned above using Cox analyses. In univariate Cox analyses, the RNA-seq-based risk score, del(17p), 1qgain, and t(12;14) subgroups as well as the ATR, CREBBP, MAP3K1, PMS1, and TP53 mutations showed a prognostic value in the CoMMpass cohort (Table 1). When the RNA-seq-based risk score and molecular subgroups were tested together (multivariate analysis), only the RNA-seq-based risk score retained prognostic value (Table 2). When the RNA-seq-based risk score and mutation were tested together, the score and PMS1 gene mutation remained independent prognostic factors in the CoMMpass cohort (Table 3).

### 3.4. RNA-Seq Risk Score Revealed New Genes Significantly Associated with MM Pathophysiology

**Figure 4:**
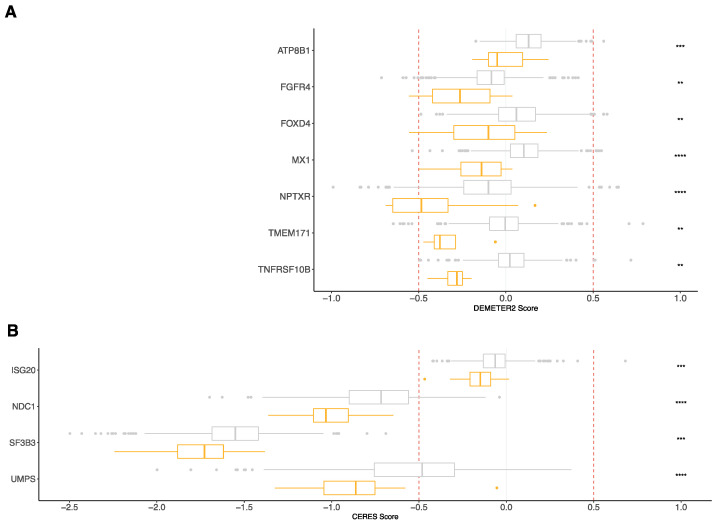
RNA-seq-based risk score revealed genes associated with MM pathophysiology. Boxplots of dependency scores calculated from the Cancer Dependency Map project (https://depmap.org) (28 September 2021). (**A**) RNAi-based viability assays in MM cell lines (n = 16) and other cancer cell lines (n = 695). (**B**) CRISPR/Cas9-based viability assays in MM cell lines (n = 20) and other cancer cell lines (n = 769). DEMETER2 and CERES scores were calculated from RNAi-based and CRISPR–Cas9 viability screens, respectively. A lower score means that a gene is more likely to be dependent in a given cell line. A score of 0 is equivalent to a gene that is not essential, whereas a score of -1 corresponds to the median of all common essential genes. Boxplots in orange represent multiple myeloma cell lines, and boxplots in grey correspond to other cancer cell lines. ** *p*-value < 0.01, *** *p*-value < 0.001, **** *p*-value < 0.0001.

We used public datasets of RNAi [33,34] and CRISPR-Cas9 [35] viability assay screening (Dependency Map data, Broad Institute, www.depmap.org) (28 September 2021) to identify the essential genes in myeloma cell lines compared to other cancer cell lines. Among the 142 genes associated with poor survival composing the RNA-seq-based risk score, we found seven genes (*ATP8B1*, *FGFR4*, *FOXD4*, *MX1*, *NPTXR*, *TMEM171,* and *TNFRSF10B*) with a significantly lower DEMETER2 score in the myeloma cell lines (n = 16) compared to other cancer cell lines (n = 695) (Figure 4A and Table 4). The CERES score of four genes (*ISG20*, *NDC1*, *SF3B3*, *UMPS*) was significantly lower in the myeloma cell lines (n = 20) compared to in the other cancer cell lines (n = 769) (Figure 4B and Table 4). These essentiality scores were calculated using the RNAi [36] and CRISPR–Cas9 [35] methods, respectively. Thus, these analyses identified essential MM genes that had not previously been considered.

### 3.5. Association between RNA-Seq-Based Risk Score and Response to Treatment

**Figure 5:**
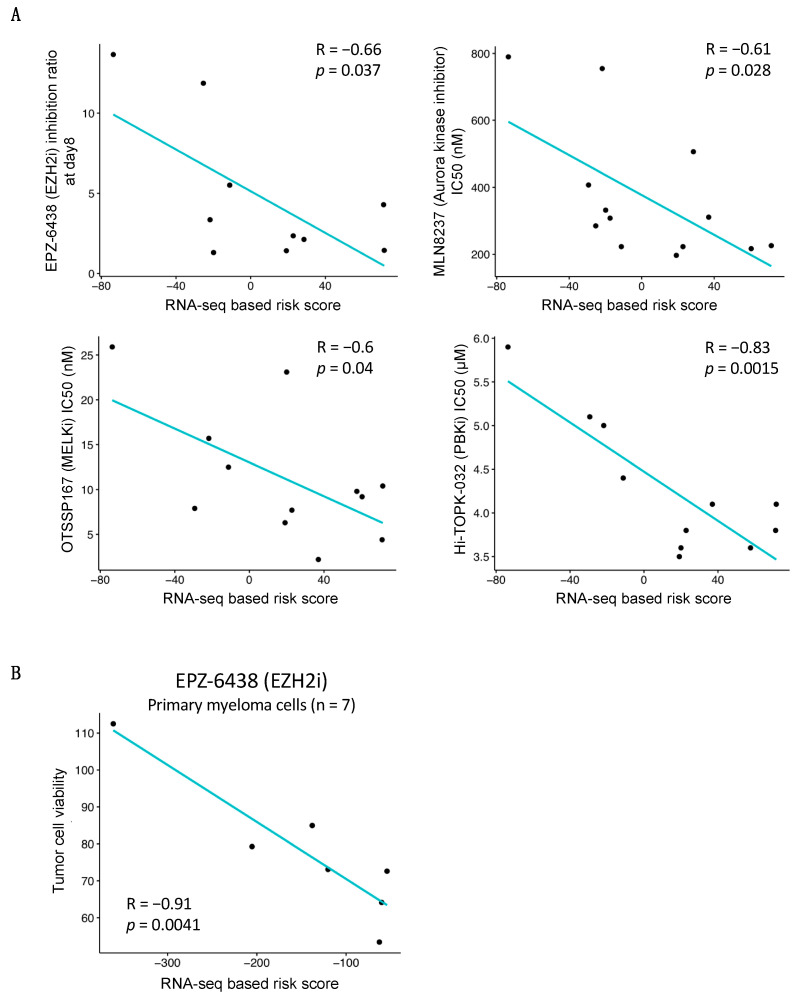
Correlation between RNA-seq-based risk score and response to targeted inhibitors. (**A**) Linear regression analysis of the inhibition ratio (treatment/control) of EPZ-6438, an EZH2 inhibitor, or the IC50 of three inhibitors of oncogene kinases (Aurora kinase inhibitor, MELKi, PBKi) in a function of the RNA-seq-based risk score in HMCLs. (**B**) RNA risk score predictions for EPZ-6438 sensitivity of primary patient myeloma cells. R represents the Pearson correlation coefficient (Pearson correlation test).

We analyzed the relationship between the RNA-seq-based risk score and HMCL response to drugs. The Spearman correlation was assessed between the RNA-seq-based risk score and IC50 for 22 different drugs [19,20,21,22,28,37]. Among them, the MM cell responses to four drugs were found to be significantly associated with the RNA-seq-based risk score (Figure 5). A significant negative correlation was observed between the risk score and the response to EZH2 (r = −0.66; *p*-value = 0.037), Aurora kinase (r = −0.61; *p*-value = 0.028), MELK (r = −0.6; *p*-value = 0.04), and TOPK/PBK (r = −0.83; *p*-value = 0.0015) inhibitors in HMCLs (Figure 5A). Moreover, as identified in the HMCLs, a significant correlation between the RNA risk score and the EZH2 inhibitor activity on the primary cells of MM patients was observed (r = −0.91; *p*-value = 0.0041) (Figure 5B). A high RNA risk score value is associated with a higher EZH2 inhibitor efficacy in patient myeloma cells. These results highlight that MM patients associated with higher RNA-seq-based risk score mays benefit from these drugs and from EZH2 inhibitor in particular.

## 4. Discussion

Here, we defined an RNA-sequencing-based transcriptomic signature with prognostic value in two independent cohorts of patients with MM. Despite a significant accumulation of knowledge related to MM drug resistance, there is a need to routinely integrate these data into clinical decision making. However, several profiling methods have been developed to provide information related to molecular classification and risk prediction. Different groups have combined GEP analysis with cytogenetics to delineate 10 different molecular subgroups with distinct prognostic values and clinical features [4,38,39]. These prognostic classifications have been associated with the clinical data and incorporated into a consensus statement by the International Myeloma Working Group (IMWG) [40]. Several groups including IFM [7], UAMS [5], our group [8], and HOVON [6] have developed microarray-based GEP prognostic signatures. More recently, large-scale clinical studies have leveraged NGS including WES and have targeted NGS panels in MM [41,42,43,44,45,46]. Microarrays are being phased out, and our study sought to validate the use of RNAseq as an alternative. There is currently a need to integrate clinically useful biomarkers to predict treatment response in association with the growing palette of anti-MM therapies that are available.

In this study, we built a 267-genes risk score using RNA-seq data. The RNA-seq-based risk score demonstrated a prognostic value in the two independent cohorts of newly diagnosed MM patients. MM patients with high-risk UAMS- or RS-microarray-defined GEP signatures demonstrated significantly higher RNA-seq-based risk score values (Figure 2B). Targeted sequencing could be used for clinical applications of the RNA-seq score where cost is important. Recently, Jang JS et al. reported a single cell RNA-seq of 597 cells derived from 15 MM patients. They developed a gene expression signature associated with MM progression that demonstrated significant prognostic value using the microarray GEP data of the APEX trial dataset [47]. We investigated the overlap between the two reported gene signatures and six common genes that were identified, including BST2, FAM214A, HBP1, ISG20, KLF6, and RAB30. The low degree of overlap could be explained by the fact that the signatures were defined using bulk RNA-seq or single-cell RNA-seq. The number of expressed genes detected from single cell RNA-seq is typically lower compared to bulk RNA-seq. The high-risk patients identified with our score were characterized by the genes involved in several major pathways implicated in MM pathophysiology, including interferon response, cell proliferation, hypoxia, IL-6 signaling pathway, stem cell genes, MYC, and epigenetic deregulation (Figure 2A). c-MYC is a key regulator in MM with deregulations related to translocations, gains and amplification, mutations in RAS genes, and MYC transcription or translation activation [48]. Hypoxia is a specific feature of MM with a significant increase of hypoxia-inducible factor-1 (HIF-1) in the bone marrow of MM tumor-bearing mice [49]. This suggests that the inhibition of HIF-1-mediated transcription may represent an interesting target in MM. Recently, we reported that chetomin, an inhibitor of HIF-1/p300 interaction, exhibits specific antitumor activity in human myeloma cell lines and in the primary MM cells from patients [50]. This approach could present therapeutic interest for high-risk patients identified with the RNA-seq risk score. IL-6 is one of the major MM growth factors [51]. Blocking IL-6 signaling was thus developed into a therapeutic approach for MM. Even if the first clinical trials did not demonstrate clear benefits, the development of IL-6 antagonism is still ongoing with clinical trials [52]. The clinical and biological role of IFN is controversial in MM. Several groups have reported that IFN-α inhibits MM cell growth [53], whereas other groups have shown that it is an MM growth factor [54]. IFN-α was used in the treatment of MM, and this was stopped due to the absence of reproducible clinical efficacy [55,56]. More recently, we reported that DNA methyltransferase inhibitors induce the overexpression of IFN-regulated genes [20]. Since high-risk MM patients identified by RNA-seq are characterized by gene signature enrichment related to DNA methylation, the identified IFN response genes could be related to epigenetic deregulations. Interestingly, high-risk RNA-seq score-defined MM patients are characterized by a significant enrichment in the genes related to stem cell genes. Genes that unrelated to the cell cycle and that are overexpressed in pluripotent, hematopoietic, and mesenchymal stem cells have been reported to be significantly overexpressed in MM in association with a poor outcome [57]. Furthermore, RNA-seq-defined high-risk patients present significant enrichment in terms of repressive epigenetic modifications, including the overexpression of the Polycomb repressive complex PRC1 and PRC2 target genes, DNMT target genes, and HDAC target genes (Figure 2A). These data underline major epigenetic remodeling in high-risk MM patients. Interestingly, a significant overlap between the PRC2 and DNA methylation target genes has been reported in MM, suggesting an overlap between these repressive chromatin marks to inactivate important MM tumor suppressor genes [21]. Transcriptional programs mediated by DNA methylation and HDAC are also associated with poor outcome and key biological deregulations [19,20]. Furthermore, a combination of epidrugs has demonstrated anti-MM cell cytotoxicity in preclinical studies [21,58]. Of particular interest, using our large cohort of MM cell lines, we found that our RNA-seq-based risk score was significantly correlated with the response to the EZH2 inhibitor. Moreover, these data were validated using primary samples from MM patients. These data suggest that MM patients with a high-risk RNA-seq score may respond to EZH2i in combination with conventional treatment. Additionally, we also found that HMCLs with high RNA-seq score values presented sensitivity to three oncogene kinase inhibitors: MELKi, TOPK/PBKi, and Aurora kinase inhibitor. Aurora A kinase inhibitor in combination with bortezomib is currently in clinical development to treat relapsed or refractory MM patients (NCT01034553) [59]. MELK and PBK were identified as being of therapeutic interest in MM [22,60]. The RNA-seq score may be of interest for patient stratification in clinical trials. The validation of these results using the primary MM cells of patients will be of interest.

Interestingly, RNA-seq-based risk score values were significantly higher in the MM cells of patients characterized by *ASXL1*, *ATM*, *BRAF*, *DIS3*, *EP300*, *FGFR3*, *KMT2B*, *LRP1B*, *MAP3K1*, *MAX*, *NOTCH2*, *NUP214*, *PRDM1*, *PTPRD*, *RB1*, *ROS1*, *SETD2*, *TP53*, *TRRAP,* and *ZFHX3* mutations compared to patients with unmutated MM cells. Among these mutated genes, *TP53*, *DIS3*, *BRAF*, *PRDM1,* are *RB1* are part of the most frequently mutated genes in MM patients [61,62]. *RB1* mutation is involved in MM pathogenesis in the same way as *BRAF* and *DIS3* [61,63,64] and is associated with a shorter outcome than *ATM* and *TP53* [65,66,67]. Furthermore, the RNA-seq-based risk score was significantly higher in MM patients with double-hit events TP53^mut^/del(17p) or FGFR3^mut^/t(4;14) compared to MM patients without double-hit events. These double-hits are associated with very aggressive disease and therapeutic resistance [68,69].

Moreover, among the genes composing the RNA-seq-based risk score and associated with bad prognosis, 11 genes were identified as significant essential MM genes, including *SF3B3*, *FGFR4*, *TNFRSF10B*, *UMPS,* and *NPTXR*. *SF3B3* encodes a subunit of the splicing factor 3b protein complex. SF3B3 regulates *EZH2* alternative splicing, and its expression is associated with poor outcome in renal cell carcinoma [70]. Moreover, the alternative splicing of *EZH2* seems to have an important role in the tumorigenesis of human renal cancer. Since EZH2 is overexpressed in myeloma cells in association with a poor outcome [21,71], it would be interesting to explore the potential role of alternative EZH2 splicing regulated by SF3B3 in plasma cell tumorigenesis and disease progression. Furthermore, the potential role of *SF3B3* in splicing regulation in MM remains unknown. The protein encoded by the *FGFR4* (fibroblast growth factor receptor 4) gene is a tyrosine kinase and cell surface receptor for fibroblast growth factors (FGF). Acting as proangiogenic and mitogenic cytokines, FGF/FGFR protects myeloma cells for oxidative stress-induced apoptosis, leading to myeloma cell survival and progression [72]. In Rhabdomyosarcoma, FGFR4-specific single-domain antibodies demonstrated a good specificity and affinity for targeting FGFR4-expressing cells and for blocking the FGF19–FGFR4–MAPK signaling axis [73]. These FGFR4 antibodies could be of therapeutic interest for high-risk MM patients who have been identified with the RNA-seq-based risk score. *TNFRSF10B*, also called *TRAIL-R2* or *DR5*, encodes a protein belonging to the TNF-receptor superfamily. The tumor necrosis factor-related apoptosis-inducing ligand (TRAIL) induces apoptosis through the activation of DR5 [74]. It was already reported that the anti-DR5 monoclonal antibody, lexatumumab, induces myeloma cell death [75]. Moreover, because DR5 is transcriptionally regulated by p53, the efficiency of lexatumumab is increased by p53, inducing stress in myeloma cells [76]. UMPS (uridine monophosphate synthetase) is the last enzyme in the novo pyrimidine biosynthetic pathway. The inhibition of UMPS by 5-aminoimidazole-4-carboxamide-1-beta-riboside treatment decreases UMP levels and leads to pyrimidine starvation and myeloma cell death [77]. These data identify pyrimidine biosynthesis as a potential molecular target for future therapeutic targeting in MM. The neuronal pentraxin receptor is a type II transmembrane protein that functions as a trans-synaptic organizer and anchors neuronal pentraxin complexes to plasma membranes [78]. The role of NPTXR remains unclear in cancers. NPTXR is associated with cancer progression and shorter survival in gastric and colorectal cancers [79,80]. Silencing NPTXR using a monoclonal Ab against NPTXR inhibits gastric cancer cell proliferation and leads to cell apoptosis [79]. It could be interesting to investigate the function of NPTXR in myeloma cells.

Despite improvement in MM patient survival due to the clinical use of novel agents, the acquisition of drug resistance remains a major limitation of MM therapy. Indeed, the great majority of MM patients relapse and eventually become resistant to all treatments. We developed an RNA-seq-based risk score that allows the identification of high-risk MM patients that may benefit from EZH2, MELK, TOPK/PBK, and Aurora kinase inhibitors. The RNA-seq-based risk score may be used to implement precision medicine strategies in MM.

## Figures and Tables

**Table 1:** Cox univariate and multivariate analyses to model overall survival in the CoMMpass cohort (n = 674 patients with MM) relative to the RNA-seq-based risk score, cytogenetic abnormalities.

	Univariate COX Analyses	
Prognostic Variable	Proportional HR	*p*-Value
RNA-seq-based risk score	8.787	<0.0001
del17p	1.609	NS
1qgain	0.958	NS
t(12;14)	1.890	NS
*ATR* ^mut^	1.780	NS
*CREBBP* ^mut^	1.419	NS
*MAP3K1* ^mut^	0.991	NS
*PMS1* ^mut^	5.013	0.006
*TP53* ^mut^	1.162	NS

**Table 2:** Cox univariate and mutations.

	Multivariate COX Analysis (Cytogenetic Abnormalities)	
Prognostic Variable	Proportional HR	*p*-Value
RNA-seq-based risk score	8.082	<0.0001
del17p	1.576	NS
1qgain	0.954	NS
t(12;14)	1.600	NS

**Table 3:** Cox univariate HR: hazard ratio.

	Multivariate COX Analysis (Mutations)	
Prognostic Variable	Proportional HR	*p*-Value
RNA-seq-based risk score	8.741	<0.0001
*ATR* ^mut^	1.805	NS
*CREBBP* ^mut^	1.436	NS
*MAP3K1* ^mut^	1.050	NS
*PMS1* ^mut^	5.099	0.006
*TP53* ^mut^	1.161	NS

**Table 4:** DEMETER2 and CERES score comparison between MM cell lines and other cancer cell lines. DEMETER2 and CERES scores were calculated from CRISPR-Cas9 and RNAi-based viability assays, respectively.

Gene	Dataset	T.Statistic	*p*-Value
*ATP8B1*	RNAi (Broad)	−4.4246306	1.18 × 10^−5^
*FGFR4*	Combined RNAi (Broad, Novartis, Marcotte)	−4.6107503	4.74 × 10^−6^
*FOXD4*	Combined RNAi (Broad, Novartis, Marcotte)	−4.8020113	1.91 × 10^−6^
*ISG20*	CRISPR (Avana) Public 20Q1	−3.949974	8.56 × 10^−5^
*MX1*	Combined RNAi (Broad, Novartis, Marcotte)	−7.990899	5.30 × 10^−15^
*NDC1*	CRISPR (Avana) Public 20Q1	−5.029908	6.15 × 10^−7^
*NPTXR*	Combined RNAi (Broad, Novartis, Marcotte)	−5.638059	2.47 × 10^−8^
*SF3B3*	CRISPR (Avana) Public 20Q1	−4.0305542	6.13 × 10^−5^
*TMEM171*	RNAi (Novartis)	−3.6151729	3.39 × 10^−3^
*TNFRSF10B*	RNAi (Novartis)	−3.996171	7.67 × 10^−5^
*UMPS*	CRISPR (Avana) Public 20Q1	−4.6553134	3.83 × 10^−6^

## Data Availability

HMCLs were authenticated according to their short tandem repeat profiling and their gene expression profiling using Affymetrix U133 plus 2.0 microarrays deposited in the ArrayExpress public database under accession numbers E-TABM-937 and E-TABM-1088. The RNA-seq data of normal B to PC differentiation are available in Gene Expression Omnibus under the accession number GSE148924.

## Supplementary Materials

The following are available online at https://www.mdpi.com/article/10.3390/jpm11100988/s1, Figure S1: Correlation between PCLI and RNA-seq based risk score, Table S1: List of the 267 prognostic genes constituting the RNA-seq based risk score.

## References

[1] Walker B.A., Leone P.E., Chiecchio L., Dickens N.J., Jenner M.W., Boyd K.D., Johnson D.C., Gonzalez D., Dagrada G.P., Protheroe R.K.M. (2010). A compendium of myeloma-associated chromosomal copy number abnormalities and their prognostic value. Blood.

[2] De Mel S., Lim S.H., Tung M.L., Chng W.-J. (2014). Implications of Heterogeneity in Multiple Myeloma. BioMed Res. Int..

[3] Bergsagel P.L., Kuehl W.M. (2005). Molecular Pathogenesis and a Consequent Classification of Multiple Myeloma. J. Clin. Oncol..

[4] Zhan F., Huang Y., Colla S., Stewart J.P., Hanamura I., Gupta S., Epstein J., Yaccoby S., Sawyer J., Burington B. (2006). The molecular classification of multiple myeloma. Blood.

[5] Shaughnessy J.D., Zhan F., Burington B.E., Huang Y., Colla S., Hanamura I., Stewart J.P., Kordsmeier B., Randolph C., Williams D.R. (2007). A validated gene expression model of high-risk multiple myeloma is defined by deregulated expression of genes mapping to chromosome. Blood.

[6] Kuiper R., Broyl A., De Knegt Y., Van Vliet M.H., Van Beers E.H., Van Der Holt B., El Jarari L., Mulligan G., Gregory W., Morgan G. (2012). A gene expression signature for high-risk multiple myeloma. Leukemia.

[7] Decaux O., Lodé L., Magrangeas F., Charbonnel C., Gouraud W., Jézéquel P., Attal M., Harousseau J.-L., Moreau P., Bataille R. (2008). Prediction of Survival in Multiple Myeloma Based on Gene Expression Profiles Reveals Cell Cycle and Chromosomal Instability Signatures in High-Risk Patients and Hyperdiploid Signatures in Low-Risk Patients: A Study of the Intergroupe Francophone du Myélome. J. Clin. Oncol..

[8] Rème T., Hose D., Theillet C., Klein B. (2013). Modeling risk stratification in human cancer. Bioinformatics.

[9] Dickens N., Walker B.A., Leone P.E., Johnson D.C., Brito J.L., Zeisig A., Jenner M., Boyd K., Gonzalez D., Gregory W.M. (2010). Homozygous Deletion Mapping in Myeloma Samples Identifies Genes and an Expression Signature Relevant to Pathogenesis and Outcome. Clin. Cancer Res..

[10] Hose D., Rème T., Hielscher T., Moreaux J., Messner T., Seckinger A., Benner A., Shaughnessy J.D., Barlogie B., Zhou Y. (2011). Proliferation is a central independent prognostic factor and target for personalized and risk-adapted treatment in multiple myeloma. Haematologica.

[11] Chng W.J., Braggio E., Mulligan G., Bryant B., Remstein E., Valdez R., Dogan A., Fonseca R. (2008). The centrosome index is a powerful prognostic marker in myeloma and identifies a cohort of patients that might benefit from aurora kinase inhibition. Blood.

[12] Jourdan M., Reme T., Goldschmidt H., Fiol G., Pantesco V., DE Vos J., Rossi J.-F., Hose D., Klein B. (2009). Gene expression of anti- and pro-apoptotic proteins in malignant and normal plasma cells. Br. J. Haematol..

[13] Shaughnessy J.D., Qu P., Usmani S., Heuck C.J., Zhang Q., Zhou Y., Tian E., Hanamura I., Van Rhee F., Anaissie E. (2011). Pharmacogenomics of bortezomib test-dosing identifies hyperexpression of proteasome genes, especially PSMD4, as novel high-risk feature in myeloma treated with Total Therapy. Blood.

[14] Stessman H., Baughn L.B., Sarver A., Xia T., Deshpande R., Mansoor A., Walsh S., Sunderland J., Dolloff N.G., Linden M. (2013). Profiling Bortezomib Resistance Identifies Secondary Therapies in a Mouse Myeloma Model. Mol. Cancer Ther..

[15] Mitra A., Mukherjee U.K., Harding T., Jang J.S., Stessman H., Li Y., Abyzov A., Jen J., Kumar S., Rajkumar V. (2015). Single-cell analysis of targeted transcriptome predicts drug sensitivity of single cells within human myeloma tumors. Leukemia.

[16] Mitra A., Harding T., Mukherjee U.K., Jang J.S., Li Y., Hongzheng R., Jen J., Sonneveld P., Kumar S., Kuehl W.M. (2017). A gene expression signature distinguishes innate response and resistance to proteasome inhibitors in multiple myeloma. Blood Cancer J..

[17] Gourzones C., Bellanger C., Lamure S., Gadacha O.C., De Paco E.G., Vincent L., Cartron G., Klein B., Moreaux J. (2019). Antioxidant Defenses Confer Resistance to High Dose Melphalan in Multiple Myeloma Cells. Cancers.

[18] Bhutani M., Zhang Q., Friend R., Voorhees P.M., Druhan L.J., Barlogie B., Sonneveld P., Morgan G., Symanowski J.T., Avalos B.R. (2017). Investigation of a gene signature to predict response to immunomodulatory derivatives for patients with multiple myeloma: An exploratory, retrospective study using microarray datasets from prospective clinical trials. Lancet Haematol..

[19] Moreaux J., Reme T., Leonard W., Veyrune J.-L., Requirand G., Goldschmidt H., Hose D., Klein B. (2013). Gene expression-based prediction of myeloma cell sensitivity to histone deacetylase inhibitors. Br. J. Cancer.

[20] Moreaux J., Rème T., Leonard W., Veyrune J.-L., Requirand G., Goldschmidt H., Hose D., Klein B. (2012). Development of Gene Expression–Based Score to Predict Sensitivity of Multiple Myeloma Cells to DNA Methylation Inhibitors. Mol. Cancer Ther..

[21] Herviou L., Kassambara A., Boireau S., Robert N., Requirand G., Müller-Tidow C., Vincent L., Seckinger A., Goldschmidt H., Cartron G. (2018). PRC2 targeting is a therapeutic strategy for EZ score defined high-risk multiple myeloma patients and overcome resistance to IMiDs. Clin. Epigenetics.

[22] de Boussac H., Bruyer A., Jourdan M., Maes A., Robert N., Gourzones C., Vincent L., Seckinger A., Cartron G., Hose D. (2020). Kinome expression profiling to target new therapeutic avenues in multiple myeloma. Haematologica.

[23] Dobin A., Davis C.A., Schlesinger F., Drenkow J., Zaleski C., Jha S., Batut P., Chaisson M., Gingeras T.R. (2013). STAR: Ultrafast universal RNA-seq aligner. Bioinformatics.

[24] Gentleman R.C., Carey V.J., Bates D.M., Bolstad B., Dettling M., Dudoit S., Ellis B., Gautier L., Ge Y., Gentry J. (2004). Bioconductor: Open software development for computational biology and bioinformatics. Genome Biol..

[25] Love M.I., Huber W., Anders S. (2014). Moderated estimation of fold change and dispersion for RNA-seq data with DESeq2. Genome Biol..

[26] Moreaux J., Hose D., Kassambara A., Reme T., Moine P., Requirand G., Goldschmidt H., Klein B. (2011). Osteoclast-gene expression profiling reveals osteoclast-derived CCR2 chemokines promoting myeloma cell migration. Blood.

[27] Moreaux J., Klein B., Bataille R., Descamps G., Maïga S., Hose D., Goldschmidt H., Jauch A., Rème T., Jourdan M. (2010). A high-risk signature for patients with multiple myeloma established from the molecular classification of human myeloma cell lines. Haematologica.

[28] Vikova V., Jourdan M., Robert N., Requirand G., Boireau S., Bruyer A., Vincent L., Cartron G., Klein B., Elemento O. (2019). Comprehensive characterization of the mutational landscape in multiple myeloma cell lines reveals potential drivers and pathways associated with tumor progression and drug resistance. Theranostics.

[29] Kassambara A., Hose D., Moreaux J., Walker B.A., Protopopov A., Reme T., Pellestor F., Pantesco V., Jauch A., Morgan G. (2012). Genes with a spike expression are clustered in chromosome (sub)bands and spike (sub)bands have a powerful prognostic value in patients with multiple myeloma. Haematologica.

[30] Requirand G., Robert N., Boireau S., Vincent L., Seckinger A., Bouhya S., Ceballos P., Cartron G., Hose D., Klein B. (2019). BrdU incorporation in multiparameter flow cytometry: A new cell cycle assessment approach in multiple myeloma. Cytom. Part B Clin. Cytom..

[31] Subramanian A., Tamayo P., Mootha V.K., Mukherjee S., Ebert B.L., Gillette M.A., Paulovich A., Pomeroy S.L., Golub T.R., Lander E.S. (2005). Gene set enrichment analysis: A knowledge-based approach for interpreting genome-wide expression profiles. Proc. Natl. Acad. Sci. USA.

[32] Liberzon A., Subramanian A., Pinchback R., Thorvaldsdóttir H., Tamayo P., Mesirov J.P. (2011). Molecular signatures database (MSigDB) 3. Bioinformatics.

[33] Tsherniak A., Vazquez F., Montgomery P.G., Weir B.A., Kryukov G., Cowley G.S., Gill S., Harrington W.F., Pantel S., Krill-Burger J. (2017). Defining a Cancer Dependency Map. Cell.

[34] Iii E.R.M., De Weck A., Schlabach M.R., Billy E., Mavrakis K.J., Hoffman G., Belur D., Castelletti D., Frias E., Gampa K. (2017). Project DRIVE: A compendium of cancer dependencies and synthetic lethal relationships uncovered by large-scale, deep RNAi screening. Cell.

[35] Meyers R.M., Bryan J.G., McFarland J.M., Weir B.A., Sizemore A.E., Xu H., Dharia N.V., Montgomery P.G., Cowley G.S., Pantel S. (2017). Computational correction of copy number effect improves specificity of CRISPR–Cas9 essentiality screens in cancer cells. Nat. Genet..

[36] McFarland J., Ho Z.V., Kugener G., Dempster J.M., Montgomery P.G., Bryan J., Krill-Burger J.M., Green T.M., Vazquez F., Boehm J.S. (2018). Improved estimation of cancer dependencies from large-scale RNAi screens using model-based normalization and data integration. Nat. Commun..

[37] Moreaux J., Bruyer A., Veyrune J.-L., Goldschmidt H., Hose D., Klein B. (2013). DNA methylation score is predictive of myeloma cell sensitivity to 5-azacitidine. Br. J. Haematol..

[38] Bergsagel P.L., Kuehl W.M., Zhan F., Sawyer J., Barlogie B., Shaughnessy J.J. (2005). Cyclin D dysregulation: An early and unifying pathogenic event in multiple myeloma. Blood.

[39] Broyl A., Hose D., Lokhorst H., de Knegt Y., Peeters J., Jauch A., Bertsch U., Buijs A., Stevens-Kroef M., Beverloo H.B. (2010). Gene expression profiling for molecular classification of multiple myeloma in newly diagnosed patients. Blood.

[40] Chng W.J., Dispenzieri A., Chim C.-S., Fonseca R., Goldschmidt H., Lentzsch S., Munshi N., Palumbo A., Miguel J.S., Sonneveld P. (2014). IMWG consensus on risk stratification in multiple myeloma. Leukemia.

[41] Kortüm K.M., Langer C., Monge J., Bruins L., Egan J.B., Zhu Y.X., Shi C.X., Jedlowski P., Schmidt J., Ojha J. (2014). Targeted sequencing using a 47 gene multiple myeloma mutation panel (M(3) P) in -17p high risk disease. Br. J. Haematol..

[42] Jiménez C., Jara-Acevedo M., Sánchez L.A.C., Castillo D., Ordóñez G.R., Sarasquete M.E., Puig N., Martínez-López J., Prieto-Conde M.I., García-Álvarez M. (2017). A Next-Generation Sequencing Strategy for Evaluating the Most Common Genetic Abnormalities in Multiple Myeloma. J. Mol. Diagn..

[43] Ryland G.L., Jones K., Chin M., Markham J., Aydogan E., Kankanige Y., Caruso M., Guinto J., Dickinson M., Prince H.M. (2018). Novel genomic findings in multiple myeloma identified through routine diagnostic sequencing. J. Clin. Pathol..

[44] Kortüm K.M., Mai E.K., Hanafiah N.H., Shi C.-X., Zhu Y.-X., Bruins L., Barrio S., Jedlowski P., Merz M., Xu J. (2016). Targeted sequencing of refractory myeloma reveals a high incidence of mutations in CRBN and Ras pathway genes. Blood.

[45] Corre J., Cleynen A., Du Pont S.R., Buisson L., Bolli N., Attal M., Munshi N., Avet-Loiseau H. (2018). Multiple myeloma clonal evolution in homogeneously treated patients. Leukemia.

[46] Rasche L., Chavan S.S., Stephens O.W., Patel P.H., Tytarenko R., Ashby C., Bauer M., Stein C., Deshpande S., Wardell C. (2017). Spatial genomic heterogeneity in multiple myeloma revealed by multi-region sequencing. Nat. Commun..

[47] Jang J.S., Li Y., Mitra A., Bi L., Abyzov A., Van Wijnen A.J., Baughn L.B., Van Ness B., Rajkumar V., Kumar S. (2019). Molecular signatures of multiple myeloma progression through single cell RNA-Seq. Blood Cancer J..

[48] Jovanović K.K., Roche-Lestienne C., Ghobrial I.M., Facon T., Quesnel B., Manier S. (2017). Targeting MYC in multiple myeloma. Leukemia.

[49] Hu J., Van Valckenborgh E., Menu E., De Bruyne E., Vanderkerken K. (2012). Understanding the hypoxic niche of multiple myeloma: Therapeutic implications and contributions of mouse models. Dis. Model. Mech..

[50] Viziteu E., Grandmougin C., Goldschmidt H., Seckinger A., Hose D., Klein B., Moreaux J. (2016). Chetomin, targeting HIF-1α/p300 complex, exhibits antitumour activity in multiple myeloma. Br. J. Cancer.

[51] Sprynski A.C., Hose D., Caillot L., Réme T., Shaughnessy J.J.D., Barlogie B., Seckinger A., Moreaux J., Hundemer M., Jourdan M. (2009). The role of IGF-1 as a major growth factor for myeloma cell lines and the prognostic relevance of the expression of its receptor. Blood.

[52] Matthes T., Manfroi B., Huard B. (2016). Revisiting IL-6 antagonism in multiple myeloma. Crit. Rev. Oncol..

[53] Arora T., Jelinek D.F. (1998). Differential myeloma cell responsiveness to interferon-alpha correlates with differential induction of p19(INK4d) and cyclin D2 expression. J. Biol. Chem..

[54] Ferlin-Bezombes M., Jourdan M., Liautard J., Brochier J., Rossi J.F., Klein B. (1998). IFN-alpha is a survival factor for human myeloma cells and reduces dexamethasone-induced apoptosis. J. Immunol..

[55] Barlogie B., Kyle R.A., Anderson K.C., Greipp P.R., Lazarus H.M., Hurd D.D., McCoy J., Jr D.F.M., Dakhil S.R., Lanier K.S. (2006). Standard Chemotherapy Compared With High-Dose Chemoradiotherapy for Multiple Myeloma: Final Results of Phase III US Intergroup Trial S9321. J. Clin. Oncol..

[56] Cunningham D., Powles R., Malpas J., Raje N., Milan S., Viner C., Montes A., Hickish T., Nicolson M., Johnson P. (1998). A randomized trial of maintenance interferon following high-dose chemotherapy in multiple myeloma: Long-term follow-up results. Br. J. Haematol..

[57] Kassambara A., Hose D., Moreaux J., Rème T., Torrent J., Rossi J.F., Goldschmidt H., Klein B. (2012). Identification of Pluripotent and Adult Stem Cell Genes Unrelated to Cell Cycle and Associated with Poor Prognosis in Multiple Myeloma. PLoS ONE.

[58] Bruyer A., Maes K., Herviou L., Kassambara A., Seckinger A., Cartron G., Rème T., Robert N., Requirand G., Boireau S. (2018). DNMTi/HDACi combined epigenetic targeted treatment induces reprogramming of myeloma cells in the direction of normal plasma cells. Br. J. Cancer.

[59] Rosenthal A., Kumar S., Hofmeister C., Laubach J., Vij R., Dueck A., Gano K., Stewart A.K. (2016). A Phase Ib Study of the combination of Aurora Kinase Inhibitor alisertib (MLN8237) and bortezomib in Relapsed or Refractory Multiple Myeloma. Br. J. Haematol..

[60] Bolomsky A., Heusschen R., Schlangen K., Stangelberger K., Muller J., Schreiner W., Zojer N., Caers J., Ludwig H. (2018). Maternal embryonic leucine zipper kinase is a novel target for proliferation-associated high-risk myeloma. Haematologica.

[61] Lohr J.G., Stojanov P., Carter S.L., Cruz-Gordillo P., Lawrence M.S., Auclair D., Sougnez C., Knoechel B., Gould J., Saksena G. (2014). Widespread Genetic Heterogeneity in Multiple Myeloma: Implications for Targeted Therapy. Cancer Cell.

[62] Chapman M.A., Lawrence M.S., Keats J., Cibulskis K., Sougnez C., Schinzel A.C., Harview C., Brunet J.-P., Ahmann G.J., Adli M. (2011). Initial genome sequencing and analysis of multiple myeloma. Nat. Cell Biol..

[63] Dao D.D., Sawyer J.R., Epstein J., Hoover R.G., Barlogie B., Tricot G. (1994). Deletion of the retinoblastoma gene in multiple myeloma. Leukemia.

[64] Weißbach S., Langer C., Puppe B., Nedeva T., Bach E., Kull M., Bargou R., Einsele H., Rosenwald A., Knop S. (2014). The molecular spectrum and clinical impact ofDIS3mutations in multiple myeloma. Br. J. Haematol..

[65] Chng W.J., Price-Troska T., Gonzalez-Paz N., Van Wier S., Jacobus S., Blood E., Henderson K., Oken M., Van Ness B., Greipp P. (2007). Clinical significance of TP53 mutation in myeloma. Leukemia.

[66] Walker B., Boyle E.M., Wardell C., Murison A., Begum D.B., Dahir N.M., Proszek P.Z., Johnson D.C., Kaiser M.F., Melchor L. (2015). Mutational Spectrum, Copy Number Changes, and Outcome: Results of a Sequencing Study of Patients With Newly Diagnosed Myeloma. J. Clin. Oncol..

[67] Chavan S.S., He J., Tytarenko R., Deshpande S., Patel P., Bailey M., Stein C.K., Stephens O., Weinhold N., Petty N. (2017). Bi-allelic inactivation is more prevalent at relapse in multiple myeloma, identifying RB1 as an independent prognostic marker. Blood Cancer J..

[68] Benard B., Christofferson A., Legendre C., Jessica A., Sara N., Jennifer Y., Daniel A., Winnie L., Sagar L., Jonathan J.K. (2017). FGFR3 Mutations Are an Adverse Prognostic Factor in Patients with t(4;14)(p16;q32) Multiple Myeloma: An Mmrf Commpass Analysis. Blood.

[69] Weinhold N., Ashby C., Rasche L., Chavan S.S., Stein C., Stephens O.W., Tytarenko R., Bauer M., Meißner T., Deshpande S. (2016). Clonal selection and double-hit events involving tumor suppressor genes underlie relapse in myeloma. Blood.

[70] Xiao H., Chen K., Xu H. (2016). MP85-05 Alternative splicing of EZH2 pre-mRNA by SF3B3 contributes to the tumorigenic potential of renal cancer. J. Urol..

[71] Pawlyn C., Bright M.D., Buros A.F., Stein C.K., Walters Z., Aronson L.I., Mirabella F., Jones J.R., Kaiser M.F., Walker B.A. (2017). Overexpression of EZH2 in multiple myeloma is associated with poor prognosis and dysregulation of cell cycle control. Blood Cancer J..

[72] Ronca R., Ghedini G.C., Maccarinelli F., Sacco A., Locatelli S.L., Foglio E., Taranto S., Grillo E., Matarazzo S., Castelli R. (2020). FGF Trapping Inhibits Multiple Myeloma Growth through c-Myc Degradation–Induced Mitochondrial Oxidative Stress. Cancer Res..

[73] Alijaj N., Moutel S., Gouveia Z.L., Gray M., Roveri M., Dzhumashev D., Weber F., Meier G., Luciani P., Rössler J.K. (2020). Novel FGFR4-Targeting Single-Domain Antibodies for Multiple Targeted Therapies against Rhabdomyosarcoma. Cancers.

[74] Kimberley F.C., Screaton G.R. (2004). Following a TRAIL: Update on a ligand and its five receptors. Cell Res..

[75] Menoret E., Bougie P.G., Geffroy-Luseau A., Daniels S., Moreau P., le Gouill S., Harousseau J.-L., Bataille R., Amiot M., Deceunynck C. (2006). Mcl-1L cleavage is involved in TRAIL-R1– and TRAIL-R2–mediated apoptosis induced by HGS-ETR1 and HGS-ETR2 human mAbs in myeloma cells. Blood.

[76] Surget S., Chiron D., Gomez-Bougie P., Descamps G., Ménoret E., Bataille R., Moreau P., Le Gouill S., Amiot M., Pellat-Deceunynck C. (2012). Cell Death via DR5, but not DR4, Is Regulated by p53 in Myeloma Cells. Cancer Res..

[77] Bardeleben C., Sharma S., Reeve J.R., Bassilian S., Frost P., Hoang B., Shi Y., Lichtenstein A. (2013). Metabolomics Identifies Pyrimidine Starvation as the Mechanism of 5-Aminoimidazole-4-Carboxamide-1-β-Riboside-Induced Apoptosis in Multiple Myeloma Cells. Mol. Cancer Ther..

[78] Lee S.-J., Wei M., Zhang C., Maxeiner S., Pak C., Botelho S.C., Trotter J., Sterky F.H., Südhof T.C. (2017). Presynaptic Neuronal Pentraxin Receptor Organizes Excitatory and Inhibitory Synapses. J. Neurosci..

[79] Kanda M., Shimizu D., Sawaki K., Nakamura S., Umeda S., Miwa T., Tanaka H., Tanaka C., Hayashi M., Iguchi Y. (2020). Therapeutic monoclonal antibody targeting of neuronal pentraxin receptor to control metastasis in gastric cancer. Mol. Cancer.

[80] Xu C., Tian G., Jiang C., Xue H., Kuerbanjiang M., Sun L., Gu L., Zhou H., Liu Y., Zhang Z. (2019). NPTX2 promotes colorectal cancer growth and liver metastasis by the activation of the canonical Wnt/β-catenin pathway via FZD6. Cell Death Dis..

