# Supplementary material for: RNA-Sequencing-Based Transcriptomic Score with Prognostic and Theranostic Values in Multiple Myeloma
Source: J Pers Med. 2021 Sep 30;11(10):988. doi: 10.3390/jpm11100988 (PMC8541503; doi:10.3390/jpm11100988)
Supplement: Supplementary file 1 [file jpm-11-00988-s001.zip › jpm-1386901-supplementary.pdf]

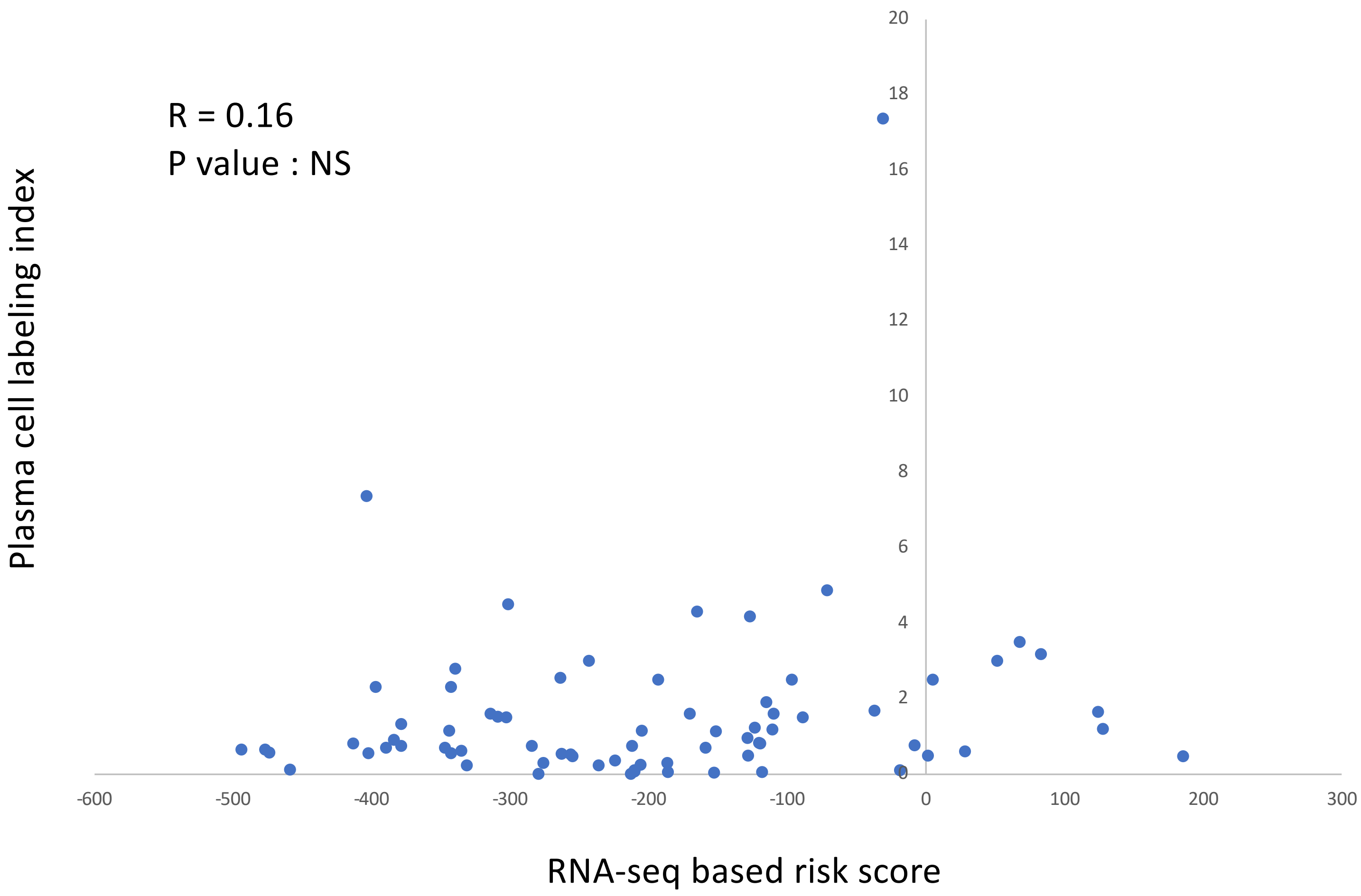

Supplementary Figure S1: Correlations between plasma cell labeling index (PCLI) and RNA-seq based risk score in the Montpellier cohort of 77 patients. NS = Non significant.
